# Comparing patterns of recent and remote Mycobacterium tuberculosis infection determined using the QuantiFERON-TB Gold Plus assay in a high TB burden setting

**DOI:** 10.1371/journal.pgph.0003182

**Published:** 2024-05-20

**Authors:** Modupe Amofa-Sekyi, Ab Schaap, Linda Mureithi, Barry Kosloff, Maina Cheeba, Bxyn Kangololo, Redwaan Vermaak, Robynn Paulsen, Maria Ruperez, Sian Floyd, Petra de Haas, Sarah Fidler, Richard Hayes, Helen Ayles, Kwame Shanaube, on behalf of the TREATS study team

**Affiliations:** 1 Zambart, Lusaka, Zambia; 2 London School of Hygiene & Tropical Medicine, London, United Kingdom; 3 Health Systems Trust, Cape Town, South Africa; 4 School of Public Health and Family Medicine, Faculty of Health Sciences, University of Cape Town, Cape Town, South Africa; 5 KNCV Tuberculosis Foundation, The Hague, Netherlands; 6 HIV Trials Unit, Imperial College London, London, United Kingdom; African Society of Laboratory Medicine, ETHIOPIA

## Abstract

One quarter of the world’s population is estimated to be infected with Mycobacterium tuberculosis. Identifying recent TB infection (TBI) offers an avenue to targeted TB preventative therapy provision, and prevention to disease progression. However, detecting recent TBI remains challenging. The QuantiFERON-TB Gold Plus assay (QFT-Plus) claims to have improved sensitivity in detecting recent TBI, by the addition of the TB2 antigen tube to the TB1 tube used in previous tests. TB2 detects CD8-mediated interferon gamma response, a potential marker of recent infection. We compared QFT-Plus TB1 and TB2 responses in individuals with recent and remote infection in high-burden settings. The Tuberculosis Reduction through Expanded Antiretroviral Treatment and TB Screening (TREATS) Project followed a cohort of adolescents and young people (AYP) aged 15–24 years in Zambia and South Africa to determine TBI incidence measured by QFT-Plus over 24 months. We categorised individuals with QTF-Plus positive result into recent and remote infection. We compared their TB1 and TB2 responses and the antigen tube differential [TB2-TB1], an indicator of CD8-activity, using logistic regression. At baseline, 3876 AYP, 1852/3876 (47.8%) were QFT-Plus positive whilst 2024/3876 (52.2%) QFT-Plus negative. Of the QFT-Plus baseline positives, 1069/1852 (57.7%) tested positive at both 12 and 24 months—remote infection. Of the QFT-Plus baseline negatives, 274/2024(13.3%) converted within a 12-month period- recent infection. TB1 and TB2 responses were higher in remote than recent infection. In recent infection, TB2 responses were greater than TB1 responses. The mean differential was 0.01 IU/ml in recent and -0.22 IU/ml in remote infection, (p = 0.145). The quantitative QFT-Plus results did not appear to reflect a marked distinction between recent and remote infection. Further analysis of the responses of infected individuals who developed disease is required to determine whether any signal in QFT-Plus results may predict progression to disease.

## Introduction

Tuberculosis (TB) is one of the leading infectious causes of death, with an estimated 1.5 million TB deaths in 2021 [1]. One quarter of the world’s population is estimated to be infected with *Mycobacterium tuberculosis* (M.tb), with 5–15% risk of developing TB disease within the first two years following infection [2, 3]. TB preventative therapy (TPT) is advocated especially in high-risk groups to reduce the risk of progression to disease [4]. However, current tests for TB infection are unable to differentiate recent from remotely infected individuals. Identifying individuals recently infected, and therefore at the highest risk of progressing to TB, may be beneficial for targeted TPT administration and scale-up [5–7].

Immunological TB assays, such as interferon gamma release assays (IGRA), tuberculin skin tests (TST) or tuberculosis antigen-based skin tests (TBST), identify infection indirectly by measuring host responses to M.tb antigens, and have been found to have similar sensitivity [8]. Currently available tests generally have a low positive predictive value to detect progression to disease [9]. A fourth generation IGRA, the QuantiFERON-TB Gold Plus assay (QFT-Plus) was designed with an aim of improving sensitivity in individuals recently exposed to TB, children and individuals with impaired immunity [10]. The QFT-Plus assay consists of four tubes—a nil tube(negative control), mitogen tube (positive control), and two M.tb antigen specific tubes -TB1 and TB2 tubes. The TB1 tube detects CD4-mediated responses whilst the TB2 tube detects both CD4- and CD8-mediated responses. CD8-mediated responses have been thought to be indicative of recent exposure to M.tb and may be considered to be a potential marker of recent TB infection [11–13]. Additionally, the differential of the interferon gamma (IFN-γ) responses between the two QFT-Plus tubes [TB2-TB1] has been proposed as an indicator of CD8-mediated activity. As the QFT-Plus assay has known intrinsic variability, a differential of ≥0.6IU/ml has been suggested as a threshold indicative of more recent infection. However, the optimal threshold is yet to be established [14–16].

In high burden TB settings, adolescents and young people (AYP) may be more likely to have more recent exposure to TB compared to older individuals who may have more years of cumulative TB exposure. AYP may therefore be a good population in which to examine recent and remote infection.

The Tuberculosis Reduction through Expanded Antiretroviral Treatment and TB Screening (TREATS) Study measured the effect of the HPTN 071 (PopART) intervention on several measures of TB burden [17–20]. TREATS was carried out in 21 communities in Zambia and Western Cape Province, South Africa. The TREATS Incidence of TB Infection Cohort Study (TREATS Infection Cohort) enrolled and followed a cohort of AYP from 2018–2020, in 14 of these communities. QFT-Plus was used to detect TB infection in the study. In this report we compare IFN-γ responses elicited by QFT- Plus in recent and remote infection.

## Methods

### Study design and setting

This study used data from the TREATS Infection Cohort in which a random sample of approximately 300 AYP (aged 15–24 years) per community were enrolled from each of 14 Arm A and C PopART communities. AYP were invited from the community to a health facility (Zambia) or a clinical research site (South Africa) where they were enrolled. Details of the cohort enrollment have been published elsewhere [19]. The study recruitment period lasted from July 12, 2018 to May 24, 2019.

Study participants aged ≥ 18 years signed an informed consent form prior to enrolment, whilst for participants ≤ 17 years both assent and parental consent were obtained. Data on socio-demographic variables, TB/HIV history, TB symptoms and risk factors were obtained using a structured questionnaire. The AYP were recruited at baseline and followed up at 12 and 24 months. Blood for QFT-Plus testing was drawn at each time point. A sputum sample for Xpert Ultra testing was obtained from TB symptomatic participants at baseline and 12 months, and from all participants at 24 months. HIV testing was offered to all participants. Participants who were HIV positive or Ultra Xpert positive were linked to care according to national guidelines.

### Laboratory testing

Blood was drawn into lithium heparin tubes and used for QFT-Plus testing carried out according to manufacturer’s instructions. The assay result was interpreted according to the manufacturer’s thresholds, with the IFN-γ response of the antigen tubes adjusted for the negative control, that is a positive QFT-Plus result being a TB1-nil or TB2-nil ≥0.35 IU/ml [10].

### Recent and remote infection

We categorized individuals into recent and remote infection based on their having a positive QFT-Plus result at specific time points. Individuals who were QFT-Plus negative at baseline and had become QFT-Plus positive at 12 months or those who were negative until 12 months and became positive at 24 months were defined as having recent infection. For these individuals, the first positive sample following a negative sample was used in analysis. Remote infection was defined as individuals who were QFT-Plus positive at baseline and remained positive for 24 months, indicating infection for at least 2 years. The QFT-Plus result of the 24-month sample was used for analysis of remote infection.

### Statistical analysis

The cohort results from both countries were analysed together. The IFN-γ responses were analysed with the QuantiFERON-Plus analysis software, which reports all responses greater than 10 IU/ml as“>10”as such values lie outside the range of values of the standard curve. The manufacturer’s positivity threshold of 0.35IU/ml was used for this analysis. We explored the characteristics of the individuals in the recent and remote infection categories.

For IFN-γ <10.0IU/ml responses, we calculated the mean TB1 and TB2 IFN-γ responses We compared the IFN-γ concentrations of the TB1 and TB2 antigen tubes between remote and recent infection using the Wilcoxon rank sum test. The Wilcoxon signed rank test was used to compare the mean TB1 and TB2 responses within the same participant samples (paired analysis) in recent and remote infection.

For IFN-γ responses >10.0IU/ml the McNemar’s test was used to compare the proportions of TB1 and TB2 in remote and recent infection.

We compared the mean antigen tube differential IFN-γ response [TB2-TB1], as an estimate of CD8 activity, between recent and remote infections as a continuous variable using the Wilcoxon rank sum test. We carried out a logistic regression analysis categorizing the mean [TB2-TB1] differential into ranges, and then looked at the association of these ranges with recency of infection.

We also carried out a sensitivity analysis restricted to the Zambian cohort (8 communities), as the optical density (OD) values from the QFT-Plus ELISA plates were available and could be used to estimate the IFN-γ concentrations from the standard curve. We assumed a linear relationship between the OD value and the IFN-γ concentration as proposed by Andrews et al. [21]. Using the previous generation assay, QuantiFERON-TB Gold-in-Tube (QGIT), they found a linear relationship between the OD values calculated from the standard curve and IFN-γ concentrations up to 12.0IU/ml. Beyond this level, extrapolated measurements from the standard curve underestimated the IFN-γ concentration. In line with this finding, we based our primary analysis on the conservative limit of 10.0 IU/ml. For the Zambian cohort we used calculated IFN-γ responses to derive the mean antigen tube differentials and compared them as a continuous variable in recent and remote infection using the Wilcoxon rank sum test. We were unable to repeat this sensitivity analysis for the South African cohort as OD values were unavailable.

### Ethics statement

Ethical approval for the TREATS study was sought from and granted by the University of Zambia Biomedical Research Ethics Committee in Zambia (ref 005-02-18), Pharma-Ethics (ref 180219727) in South Africa and the London School of Hygiene and Tropical Medicine Ethics Committee (ref14905)

Written voluntary informed consent for all study procedures was obtained for eligible participants aged 18 years and above. Written parental consent and participant assent were obtained for participants aged 15–17 years.

## Results

**Fig 1:**
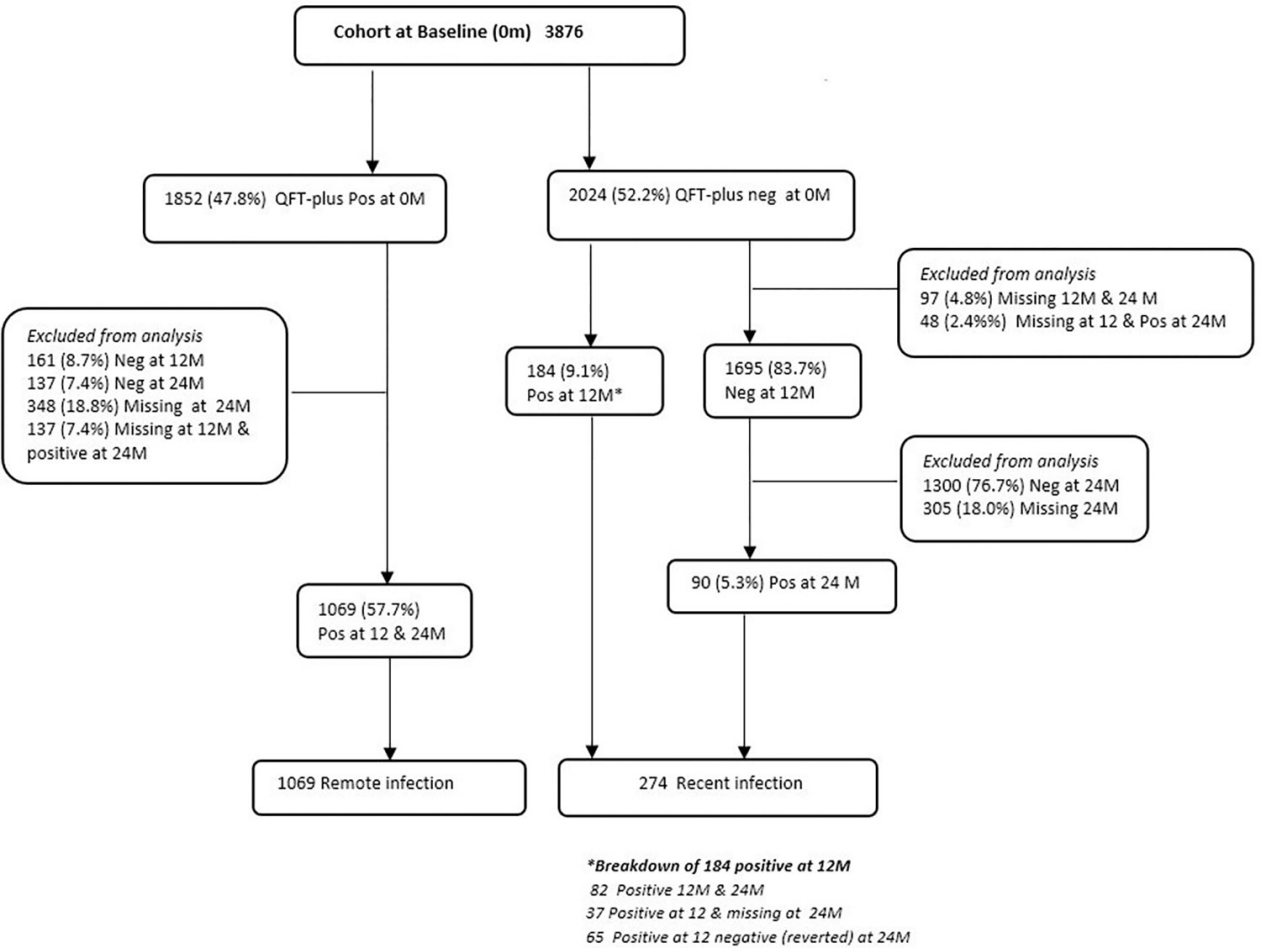
Flow chart of study participants in recent and remote infection.

At baseline, 2195 AYP in Zambia and 1682 AYP in South Africa were enrolled into the TREATS Infection cohort, with a total cohort size of 3876 AYP (Fig 1). Of these, 1852/3876 (47.8%) were QFT-Plus positive and 2024/3876 (52.2%) QFT-Plus negative at baseline. Disaggregation of QFT-Plus positivity at baseline by country showed 34.3% positivity in Zambia and 64.7% positivity in South Africa [22]. Of those QFT-Plus positive at baseline, 1069/1852 (57.7%) tested positive at 12 and 24 months and were defined as having remote infection at 24 months. Of the individuals who were QFT-Plus negative at baseline 274/2024 (13.3%) converted within a 12-month period and were defined as recent infection.

Distributions of socio-demographic characteristics including age, sex, country, HIV status, household contacts, alcohol use and smoking status, were broadly similar among the AYP in both recent and remote infection categories (Table 1). However, the Zambian cohort contributed 151/274 (55.1%) of individuals with recent infection but only 439/1069 (41.0%) of individuals with remote infection. The South African cohort contributed 630/1069 (58.9%) of the remote infections but only 123/274 (44.9%) of the recent infections (Table 1).

**Table 1:** Demographic characteristics of participants (Overall N = 1343).

Characteristic	Remote Infection	(%)	Recent infection	(%)
overall	1069	79.6%	274	20.4%
**Sex**				
Male	465	43.5.0%	130	47.5%
Female	604	56.5%	144	52.6%
**Country**				
South Africa	630	58.9%	123	44.9%
Zambia	439	41.0%	151	55.1%
**Age**				
15–16	238	22.3%	77	28.1%
17–18	229	21.4%	67	24.5%
19–20	221	20.7%	49	17.9%
21–22	194	18.2%	46	16.8%
23–24	187	17.5%	35	12.8%
**HIV status** *				
HIV negative	811	75.9%	211	77.0%
HIV positive	31	2.9%	8	2.9%
**Household contacts (HHC)** **				
No HHC	885	82.8%	237	86.5%
HHC1	136	12.7%	26	9.5%
HHC2	24	2.3%	5	1.8%
HHC3	17	1.6%	5	1.8%
**Alcohol Use**				
Never	660	61.0%	192	69.8%
Monthly	208	14.1%	40	14.5%
2–4 times a month	154	20.7%	32	11.6%
5 or more times a month	47	4.1%	11	4.0%
**Smoking Status**				
Non-smoker	793	74.2%	211	77.1%
Ex-smoker	35	3.3%	8	2.9%
Current smoker	241	22.5%	55	20.0%

*Missing values 282/1349 for HIV status not shown

**Missing values 7/1343 for HHC not shown

** HHC1: Household contacts with past history of TB, HHC2: Household contacts currently on TB Treatment, HHC3: Household contacts with past history of TB and currently on TB Treatment

### TB1 and TB2 IFN-γ responses

**Fig 2:**
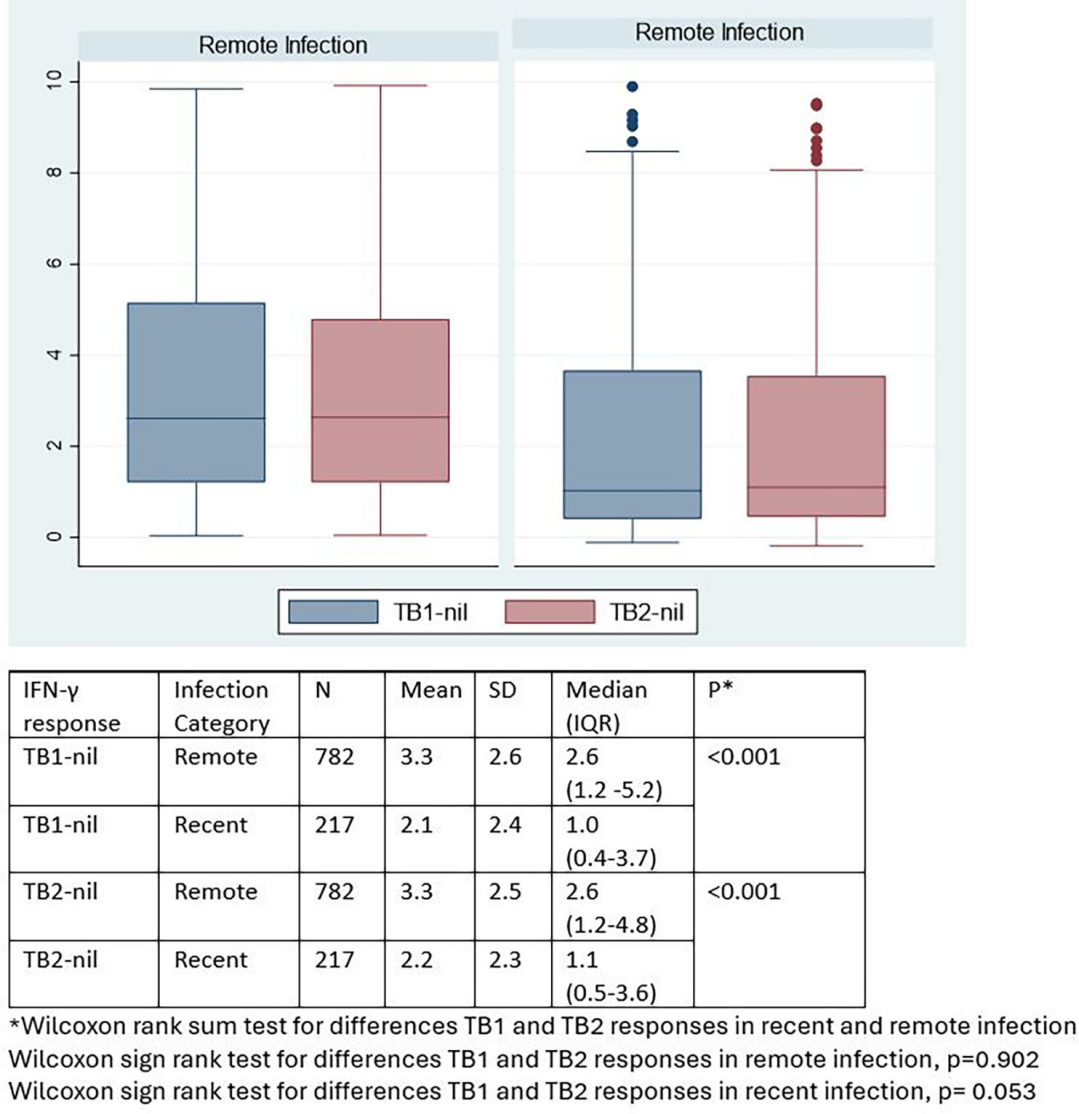
IFN-γ responses in recent and remote infection among participants with IFN-γ responses < 10 IU/ml.

Both mean TB1 and TB2 IFN-γ responses were higher in remote compared to recent infection (Table 2). The mean TB1 IFN-γ response in remote infection was 3.3IU/ml compared to 2.1IU/ml in recent infection. The mean TB2 IFN-γ response in remote infection was 3.3IU/ml compared to 2.2 IU/ml in recent infection, with strong evidence of a difference in the mean IFN-γ responses in recent and remote infection (Wilcoxon rank sum p<0.001) (Fig 2). In recent infection, the mean TB2 response (2.2IU/ml) was marginally higher than the TB1 response (2.1IU/ml) within the same participants’ samples with limited evidence of a difference, Wilcoxon signed rank p = 0.053. There was no evidence of a difference between the TB1 and TB2 responses in the same participants’ samples in remote infection (Wilcoxon signed rank test p = 0.902) (Fig 2).

**Table 2:** Numbers of TB1 and TB2 responses of participants with IFN-γ responses < 10 IU/ml.

		Remote infection				Recent infection				
		TB1 IFN-γ responses		TB2 IFN-γ responses			TB1 IFN-γ responses		TB2 IFN-γ responses	
Characteristic	N	IFN-γ responses < 10 IU/ml (%)	Mean IFN-γ concentration IU/ml	IFN-γ responses < 10.0 IU/ml (%)	Mean IFN-γ concentration IU/ml	N	IFN-γ responses < 10 IU/ml (%)	Mean IFN-γ concentration IU/ml	IFN-γ responses < 10 IU/ml (%)	Mean IFN-γ concentration IU/ml
**overall**	1069	810 (75.7%)	3.43	819 (76.6%)	3.49	274	230 (83.9%)	2.20	220 (80.3%)	2.20
**Sex**										
Male	465	359 (77.2%)	3.24	357 (76.8%)	3.28	130	103 (79.2%)	2.25	100 (76.9%)	2.42
Female	604	451 (74.7%)	3.57	462 (76.5%)	3.64	144	127 (88.2%)	2.08	120 (83.3%)	2.09
**Country**										
South Africa	630	439 (69.7%)	3.29	445 (70.6%)	3.32	123	100 (81.3%)	2.39	96 (78.0%)	2.21
Zambia	439	371 (84.5%)	3.59	374 (85.2%)	3.69	151	130 (86.1%)	2.04	124 (82.2%)	2.19
**Age**										
15–16	238	178 (74.8%)	3.37	181 (76.1%)	3.39	77	62 (80.5%)	2.30	59 (76.6%)	2.23
17–18	229	170 (74.%)	3.58	175 (76.4%)	3.60	67	55 (82.1%)	2.10	53 (79.1%)	2.28
19–20	211	168 (79.6%)	3.60	172 (81.5%)	3.59	49	42 (85.7%)	2.49	39 (80.0%)	2.34
21–22	194	150 (77.3%)	3.61	147 (75.8%)	3.60	46	40 (87.0%)	2.02	38 (82.6%)	2.05
23–24	187	144 (77.3%)	3.20	144 (77.0%)	3.24	35	31 (88.6%)	1.99	31 (88.6%)	2.00
**HIV status** *										
HIV negative	811	630 (77.7%)	3.53	638 (78.7%)	3.57	211	180 (85.3%)	2.15	172 (81.5%)	2.26
HIV positive	341	29 (8.5%)	2.81	28 (8.2%)	2.82	8	8 (100%)	2.47	8 (100%)	2.66
**Household contacts (HHC)** **										
No HHC	855	668 (78.1%)	3.43	681 (79.6%)	3.53	237	198 (83.5%)	2.22	189 (79.7%)	2.21
HHC1	136	100 (73.5%)	3.51	97 (97.0%)	2.51	26	22 (84.6%)	1.85	22 (84.6%)	1.94
HHC2	24	22 (91.7%)	3.44	21 (87.5%)	3.56	5	5 (100%)	3.11	4 (80%)	3.45
HHC3	17	14 (82.4%)	2.67	14 (82.4%)	3.10	5	4 (80.0%)	2.12	4 (80.0%)	2.30
**Alcohol Use**										
Never	660	507 (76.8%)	3.49	520 (78.8%)	3.57	191	158 (82.7%)	2.16	151 (79.1%)	2.15
Monthly	208	145 (69.7%)	3.38	144 (69.2%)	3.38	40	33 (82.5%)	2.69	31 (77.5%)	2.72
2–4 times a month	154	122 (79.2%)	3.23	122 (79.2%)	3.36	32	28 (87.5%)	2.05	27 (73.0%)	2.17
5 or more times a month	47	36 (76.6%)	3.36	33 (70.2%)	3.14	11	11 (100%)	1.62	11 (100%)	1.47
**Smoking Status**										
Non-smoker	793	600 (75.7%)	3.41	615 (77.5%)	3.50	211	175 (82.9%)	2.26	166 (78.7%)	2.25
Ex-smoker	35	31 (88.5%)	4.15	30 (85.7%)	4.20	8	7 (87.5%)	2.04	7 (87.5%)	2.21
Current smoker	241	179 (74.3%)	3.37	174 (72.2%)	3.32	55	48 (87.2%)	2.00	47 (85.5%)	2.02

*Missing values 282/1343 for HIV status not shown; **Missing values 8/1343 for HHC not shown

** HHC1: Household contacts with past history of TB, HHC2: Household contacts currently on TB Treatment, HHC3: Household contacts with past history of TB and currently on TB Treatment

We conducted a sensitivity analysis examining the TB1 and TB2 IFN-γ responses in recent and remote infections stratified by country and observed the same pattern in magnitude of responses. (S1 and S2 Figs). In an additional sensitivity analysis, we examined the effect of excluding participants in the recent infection category who were QFT-Plus negative at baseline, QFT-Plus positive at 12M, and became QFT-Plus negative at 24M, known as “reverters” (65/274) (Fig 1). Reversion typically may occur if the IFN-γ responses are weakly positive [23]. However, even after excluding the reverters from the analysis we observed the same pattern in IFN-γ responses (S3 Fig).

Among participants with recent and remote infection, 344/1343 (26.5%) had either the TB1 or TB2 IFN-γ responses being >10.0 IU/ml and as such, we were unable to calculate the mean TB1 and TB2 IFN-γ responses for these participants (S1 Table). There was generally a greater proportion of such IFN-γ responses in remote as compared to recent infection. Among recent infections, there were greater proportions of TB2 IFN-γ responses >10.0 IU/ml as compared to TB1 IFN-γ responses, McNemar test p = 0.021 (S1 Table).

### Differential antigen tube responses (TB2-TB1)

**Fig 3:**
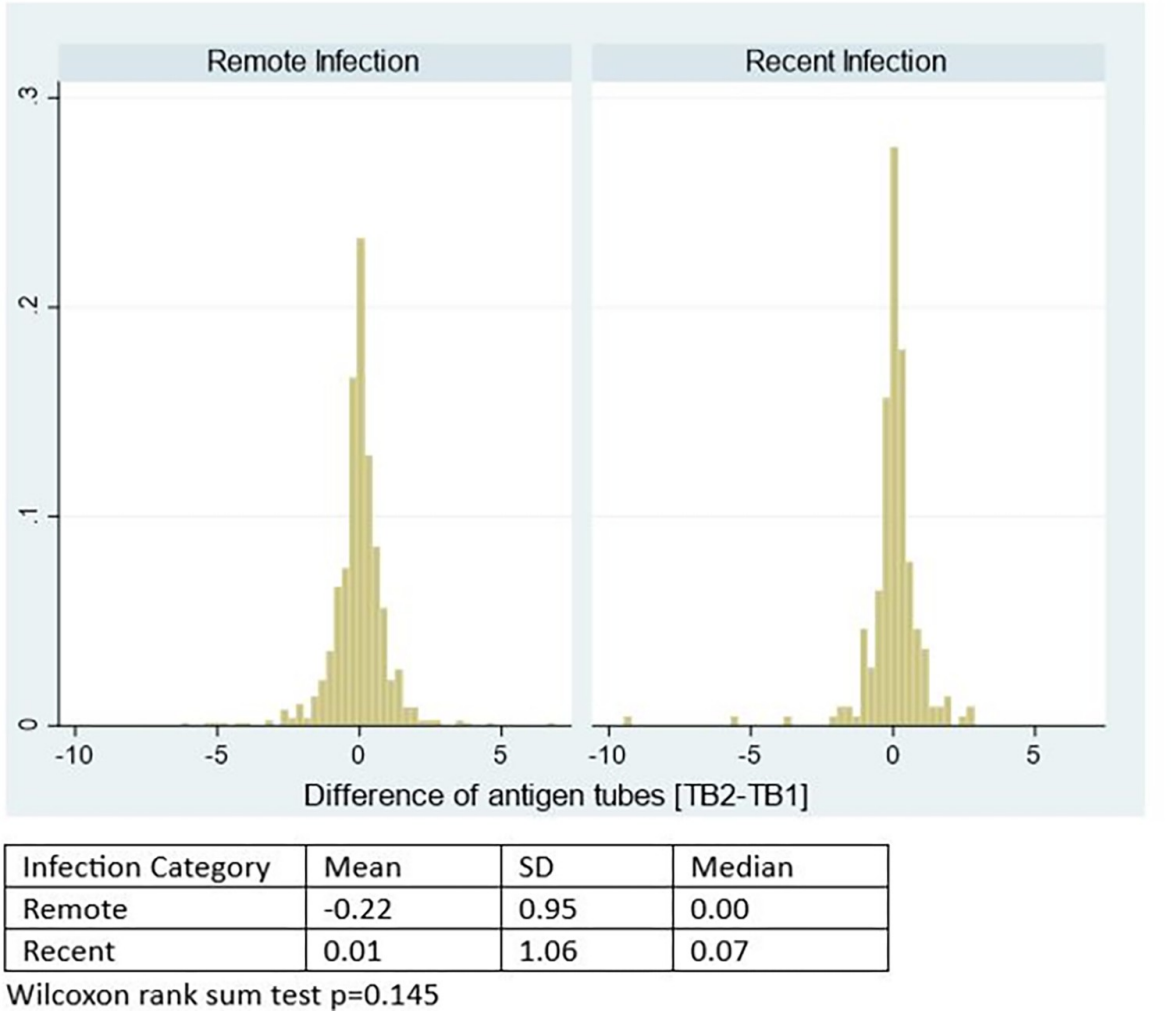
Histogram of the difference of antigen tubes (TB2-TB1) as a continuous variable among participants with IFN-γ responses < 10 IU/ml.

These responses were analysed for cohort participants with IFN-γ responses <10 IU/ml in the cohort. When looking at these responses as a continuous variable, there was a greater mean [TB2-TB1] differential in recent infection 0.01 IU/ml IU/ml compared to -0.22 IU/ml, in remote infection (Fig 3). This difference was however not significant (p = 0.145). Logistic regression analysis showed no evidence of association between recent infection and various [TB2-TB1] differential categories (Table 3). However, a sensitivity analysis carried out among the Zambian cohort using calculated IFN-γ responses (therefore including participants with responses >10 IU/ml) showed a mean [TB2-TB1] differential in recent infection of 0.45 IU/ml, which was significantly higher than in remote infection -0.05 IU/ml (p = 0.015) (S4 Fig).

**Table 3:** Distributions of categories of the antigen tube differentials (TB2-TB1) excluding IFN-γ responses ≥10.0 IU/ml.

[TB2-TB1] IU/ml categories	N	Remote infection n (%)	Recent infection n (%)	OR (95%CI)	p
-20.00 to -3.01	11	8 (72.7%)	3 (27.3%)	1.80 (0.43–7.59)	0.179
-3.00 to -1.01	76	64 (84.2%)	12 (15.8%)	0.90 (0.39–2.06)	
-1.00 to -0.61	78	67 (85.9%)	11 (141%)	0.79 (0.33–1.84)	
-0.60 to 0.59	666	508 (76.3%)	158 (23.7%)	1	
0.60 to 1.00	87	72 (82.8%)	15 (17.2%)	1.49 (0.83–2.67)	
1.01 to 3.00	76	58 (76.3%)	18 (23.7%)	1.49 (0.69–3.21)	
3.01 to 20.00	5	5 (100%)	0 (0%)	NA	
Total	999	782 (78.3%)	217 21.7%)		

## Discussion

We examined the quantitative QFT-Plus responses in a cohort of AYP who, according to their QFT-Plus results over the course of follow-up, could be categorized into recent or remote infection. We found that both TB1 and TB2 IFN-γ responses were higher in remote as compared to recent infection. In recent infection, TB2 IFN-γ responses were greater than TB1 responses. The [TB2-TB1] differential was higher in recent compared to remote infection although this difference was not statistically significant. We did not observe any associations between categories of the antigen tube differential and recency of infection.

As the TB2 tube of QFT-Plus which elicits CD8-meditated responses, was introduced into the assay to enhance detection of recent exposure to M.tb, we had expected greater TB2 responses in recent infection compared to remote infection. We found in recent infection, the mean TB2 response (2.2IU/ml) was marginally higher than the TB1 response (2.1IU/ml) with limited evidence of a difference, p = 0.053 (Fig 2). Our findings are similar to a study in Japan looking at the role of CD8 responses in 412 recent TB contacts screened with QFT-Plus for infection. They found 7.5% with overall QFT-Plus positivity, 6.3% TB1 positivity and 7.2% TB2 positivity though this latter difference was not statistically significant [24].

Possible reasons for higher TB1 and TB2 IFN-γ responses in remote infection group as compared to recent infection in our study could be as follows. In participants living in extremely high prevalence communities such as South Africa, the participants in the remote infection category may have been re-infected or exposed to higher force of infection during the follow-up period. It is also possible that remotely infected participants do just have higher IFN-γ responses.

An assessment of the risk factors associated with the baseline prevalence of infection of this cohort showed age, having a household contact and alcohol consumption in Zambia as associations but there were no associations in South Africa [22]. In both countries, social mixing patterns were explored but were not found to be associated with prevalent infection in either of the countries.

The QFT-Plus assay was developed to have improved sensitivity in immunocompromised individuals such as people living with HIV (PLHIV) and in those recently exposed to M.tb. In both of these groups we noted similar patterns in IFN-γ responses as found in the general population with higher TB2 compared to TB1 responses in the recent infection group.

Depending on the level of immunosuppression in PLHIV, there may be a reduction in the CD4 count with possibly diminished CD4 responses. The QFT-Plus assay mainly shows CD4-mediated responses in the TB1 tube, with both CD4- and CD8-mediated responses being elicited in the TB2 tube. Secondly, as CD8 responses are reportedly more prominent in recent infection, one would expect the TB2 response to be greater than TB1 response among those recently infected and this was observed. Therefore, our results may imply that the assay functioned adequately in PLHIV. However, as the PLHIV subgroup were just under 3% of the cohort, we had limited power to assess this.

Assuming that household contact with a current history of TB can be taken as a proxy of recent exposure, while household contact with individuals with a past history of TB is proxy of remote exposure; we might expect to find greater TB2 responses in recent exposure compared to remote exposure. However, we found TB2 responses exceeded TB1 responses in both groups of household contacts. This may indicate that the QFT-Plus assay has poor discrimination between recent and remote infection.

In an IGRA precision study, Metcalfe and colleagues carried out repeated IGRA testing using the QGIT on samples from the same patient [16]. They found substantial variability in the IFN-γ responses of ± 0.24 IU/ml when the initial IFN-γ response was in the range 0.25–0.80 IU/ml. They suggested that IFN-γ responses <0.59IU/ml should be interpreted cautiously. Some authors have since taken a meaningful differential to be greater than 0.6IU/ml [13, 15]. As the antigen tube differential may represent CD8 mediated activity, which is thought to be a feature of recent infection, we may expect to observe [TB2-TB1] >0.6 IU/ml among those recently infected.

We identified four previous studies, all carried out in low or intermediate burden settings, that examined the associations between a TB2-TB1>0.6IU/ml and recent infection. Three studies showed an association, and one did not.

In a contact tracing study among 119 contacts of TB patients in Italy, Barcellini and colleagues used both QFT-Plus and QFT-GIT to investigate TBI [15]. Sleeping in the same room as a TB patient (a proxy for increased exposure to M.tb) was found to show a strong association with TB2-TB1>0.6IU/ml (OR 4.34, 95% CI 1.37–13.81), p = 0.013 as compared to sleeping in a different house to an index case (OR 0.51 (0.06–4.52) p = 0.545).

In a laboratory verification study of the QFT-Plus assay conducted across 16 laboratories in Belgium and the Netherlands, samples from 1031 participants were analysed. The indication for testing included screening prior to immunotherapy, testing for TB infection as part of differential diagnosis, occupational health services screening for health care workers in contact with TB patients or exposed to materials infected with TB, and TB contact investigation. Among the various indications for testing, it was noted that samples from TB contact investigations and periodic occupational health screening, both seemingly indicative of recent infection, were more likely to have a differential antigen tube response [TB2-TB1]>0.6IU/ml, as compared to other indications, (p = 0.029) [13].

A study carried out in an outpatient clinic in Portugal, retrospectively evaluated 1165 patients who were being screened for TB infection with the QFT-Plus assay over one year with the aim of evaluating CD8-mediated IFN-γ responses as a marker of recent M tb infection [14]. Patients with recent contact (M tb. exposure within the past year) were compared to those who were being screened with no history of contact. Those with recent contact were found to be more likely to have a tube differential [TB2-TB1] >0.6IU/ml as compared to those with no history of contact OR 1.80 (95% CI 1.14–2.84), p = 0.012.

A multicenter retrospective study mainly carried out in European settings evaluated QFT-Plus IFN-γ responses among different groups stratified into high, intermediate, and low risk of recent TB exposure. The study was conducted among a total of 686 adults who were either contacts of microbiologically confirmed-TB patients (high risk), asylum seekers or people from abroad (intermediate risk) and individuals screened for TB infection prior to immune-suppressant therapy for immune-mediated inflammatory disease (low risk). There was a lack of association between an antigen tube differential ≥ 0.6IU/ml and the likelihood of recent TB exposure both in unadjusted and adjusted analyses [25].

In all of the studies above, there was generally good correlation between individual TB1 and TB2 antigen tube positivity, a trend which has been noted in several studies which utilized the assay to determine infection [22, 26–28].

To the best of our knowledge, there have been no longitudinal studies in high burden settings which have analysed the antigen tube differentials for this assay. We identified one cross-sectional study which reported on individual antigen tube positivity. That study was conducted in Uganda among pregnant woman attending an antenatal clinic at a national referral hospital. The QFT-Plus assay was used to determine prevalence of TB infection using a positivity threshold of 0.35IU/ml. The study reported 37.9% (99/261) had a positive QFT-Plus result, with 11.1% (11/99) positive on TB1 alone and 18.2% (18/99) positive on TB2 alone [29].The authors did not categorize the infections according to recent or remote infection.

Our study is unique in characterizing participants in terms of recent or remote infection, which allowed us to directly compare QFT-Plus results quantitatively in individuals with long-term positive results and those who had recently converted from negative to positive. The analysis of the TB1 and TB2 responses of cohort members who progressed to TB will also provide additional data on whether there are signals the assay results may be predictive of progression to disease.

### Limitations and strengths

A strength of our study was the ability to assess QFT-Plus responses in individuals with clearly defined recent and remote TB infection status. As we followed-up participants over a two-year period, we were able to categorize individuals accurately as having a positive result for more than two years, and therefore being remotely infected. Secondly, the large size of our cohort provided us with sufficient power and precision to draw reliable conclusions. Our analysis may have been affected by participants who missed one or both follow-up visits at 12 and 24 months, but to mitigate against this we excluded these individuals from the analysis. A limitation is that we assume that conversion of the test result from negative to positive represents recent infection while persistent positivity indicates remote infection, but in high burden settings those who were persistently positive may have been a mix of remote infections and re-infections.

## Conclusion

Among this cohort of adolescents and young people in a high TB burden setting, we found differences in QFT-Plus responses between recent and remote infection, with TB1 and TB2 IFN-γ responses higher in remote infection as compared to recent infection. In recent infection, TB2 IFN-γ responses were greater than TB1 responses. Overall, the quantitative QFT-Plus assay results did not appear to reflect a marked distinction between recent and remote infection and may be of limited clinical application. Further analysis of the QFT-Plus responses of the cohort members who progressed to TB is required to determine if there is any signal in the QFT-plus assay results that may predict risk of progression to disease.

## Supporting information

S1 Table The number of TB1 and TB2 responses of participants with IFN-γ responses >10.0 IU/ml. (DOCX)

S1 Fig IFN-γ responses in recent and remote infection among participants with response < 10IU/ml (Zambia). (TIF)

S2 Fig IFN-γ responses in recent and remote infection among participants with response < 10IU/ml (South Africa). (TIF)

S3 Fig IFN-γ responses in recent and remote infection among participants with IFN-γ responses < 10IU/ml excluding participants with recent infection who at reverted 24M. (TIF)

S4 Fig Histogram of the difference of antigen tubes for calculated IFN-γ responses among Zambia cohort. (TIF)
