# Supplementary material for: Comparing patterns of recent and remote Mycobacterium tuberculosis infection determined using the QuantiFERON-TB Gold Plus assay in a high TB burden setting
Source: PLOS Glob Public Health. 2024 May 20;4(5):e0003182. doi: 10.1371/journal.pgph.0003182 (PMC11104639; doi:10.1371/journal.pgph.0003182)
Supplement: S1 Table — (DOCX) [file pgph.0003182.s001.docx]

**S1** **Table :** **The number of TB1 and TB2 responses of participants with IFN-γ responses >10.0 IU/ml**

|  | **Remote infection** | | | | | **Recent infection** | | | | |
| --- | --- | --- | --- | --- | --- | --- | --- | --- | --- | --- |
|  |  | **TB1** | | **TB2** | |  | **TB1** | | **TB2** | |
| **Characteristic** | **N** | **n** | **n/N**  **(%)** | **n** | **n/N**  **(%)** | **N** | **n** | **n/N**  **%** | **n** | **n/N**  **%** |
| **Overall^ǂ^** | 1069 | 259 | 24.2 | 250 | 23.4 | 274 | 44 | 16.1 | 54 | 19.7 |
| **Sex** |  |  |  |  |  |  |  |  |  |  |
| Male | 465 | 106 | 22.8 | 108 | 23.2 | 130 | 27 | 20.8 | 30 | 80.0 |
| Female | 604 | 153 | 25.3 | 142 | 23.5 | 144 | 17 | 11.8 | 24 | 16.7 |
| **Country** |  |  |  |  |  |  |  |  |  |  |
| South Africa | 630 | 191 | 30.3 | 185 | 29.4 | 123 | 23 | 18.7 | 27 | 22.0 |
| Zambia | 439 | 68 | 15.5 | 65 | 14.8 | 151 | 21 | 13.9 | 27 | 17.9 |
| **Age** |  |  |  |  |  |  |  |  |  |  |
| 15-16 | 238 | 60 | 25.2 | 57 | 23.9 | 77 | 15 | 19.5 | 18 | 23.4 |
| 17-18 | 229 | 59 | 25.8 | 54 | 23.6 | 67 | 12 | 17.9 | 14 | 20.9 |
| 19-20 | 211 | 53 | 25.1 | 49 | 23.2 | 49 | 7 | 14.2 | 10 | 20.4 |
| 21-22 | 194 | 44 | 22.9 | 47 | 24.2 | 46 | 6 | 13.0 | 8 | 9.3 |
| 23-24 | 187 | 43 | 23.0 | 43 | 23.0 | 35 | 4 | 11.4 | 4 | 11.4 |
| **HIV status*** |  |  |  |  |  |  |  |  |  |  |
| HIV negative | 811 | 181 | 22.3 | 173 | 21.3 | 211 | 31 | 14.7 | 39 | 18.4 |
| HIV positive | 341 | 2 | 0.60 | 3 | 0.90 | 8 | 0 | 0 | 0 | 0 |
| **Household contacts (HHC)**** |  |  |  |  |  |  |  |  |  |  |
| No HHC | 855 | 217 | 25.4 | 204 | 23.9 | 237 | 39 | 16.5 | 48 | 20.3 |
| HHC1 | 136 | 36 | 26.4 | 39 | 28.7 | 26 | 4 | 15.4 | 4 | 15.4 |
| HHC2 | 24 | 2 | 8.3 | 3 | 12.5 | 5 | 0 | 0 | 1 | 20.0 |
| HHC3 | 17 | 3 | 17.6 | 3 | 17.6 | 5 | 1 | 20.0 | 1 | 20.0 |
| **Alcohol Use** |  |  |  |  |  |  |  |  |  |  |
| Never | 660 | 153 | 23.2 | 140 | 21.2 | 191 | 33 | 17.3 | 40 | 20.9 |
| Monthly | 208 | 63 | 30.2 | 64 | 30.7 | 40 | 7 | 17.5 | 9 | 22.5 |
| 2-4 times a month | 154 | 32 | 20.8 | 32 | 20.8 | 32 | 4 | 12.5 | 5 | 15.6 |
| 5 or more times a month | 47 | 11 | 23.4 | 14 | 29.8 | 11 | 0 | 0 | 0 | 0 |
| **Smoking Status** |  |  |  |  |  |  |  |  |  |  |
| Non-smoker | 793 | 193 | 24.3 | 178 | 22.4 | 211 | 36 | 17.1 | 45 | 21.3 |
| Ex-smoker | 35 | 4 | 11.4 | 5 | 14.3 | 8 | 1 | 12.5 | 1 | 12.8 |
| Current smoker | 241 | 62 | 27.8 | 67 | 27.8 | 55 | 7 | 12.7 | 8 | 14.5 |

ǂMcNemar’s test for differences in proportions of TB1 and TB2 IFN-γ responses >10.0 IU/ml in remote infection p=0.267

McNemar’s test for differences in proportions of TB1 and TB2 IFN-γ responses >10.0 IU/ml in recent infection p= 0. 0.021

*Missing values 329/1481 for HIV status not shown; **Missing values 7/1481 for HHC not shown

** HHC1: Household contacts with past history of TB, HHC2: Household contacts currently on TB Treatment , HHC3: Household contacts with past history of TB and currently on TB Treatment
