# Supplementary figures and images for: Comparing patterns of recent and remote Mycobacterium tuberculosis infection determined using the QuantiFERON-TB Gold Plus assay in a high TB burden setting
Source: PLOS Glob Public Health. 2024 May 20;4(5):e0003182. doi: 10.1371/journal.pgph.0003182 (PMC11104639; doi:10.1371/journal.pgph.0003182)

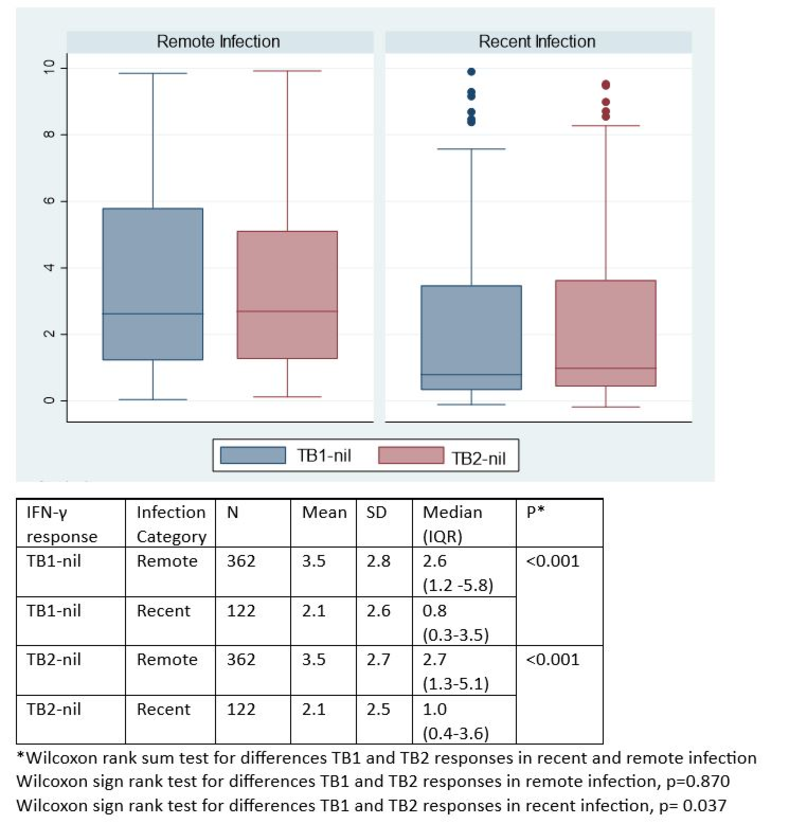

Supplement: S1 Fig — (TIF) [file pgph.0003182.s002.tif]

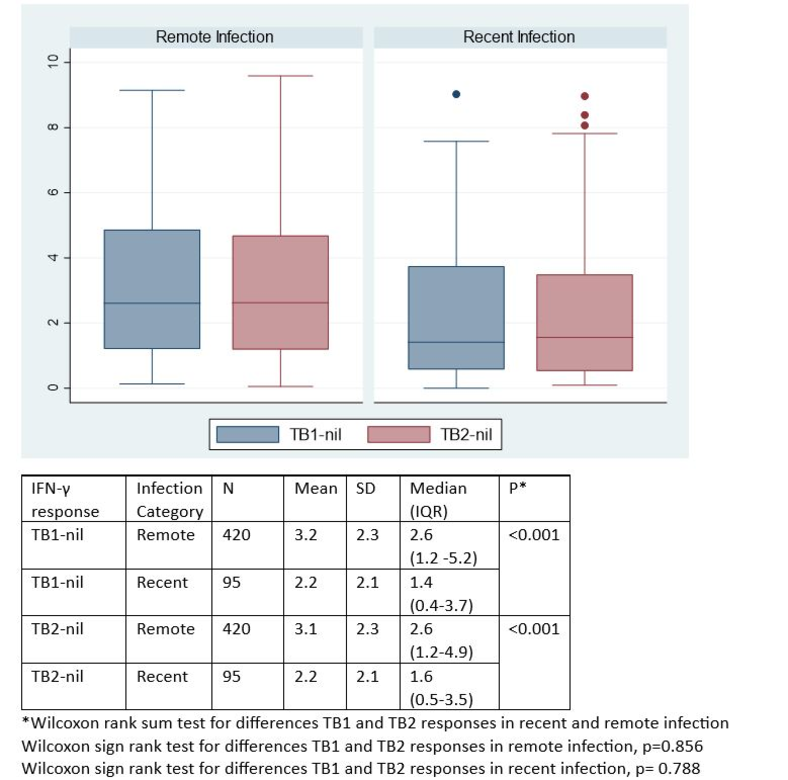

Supplement: S2 Fig — (TIF) [file pgph.0003182.s003.tif]

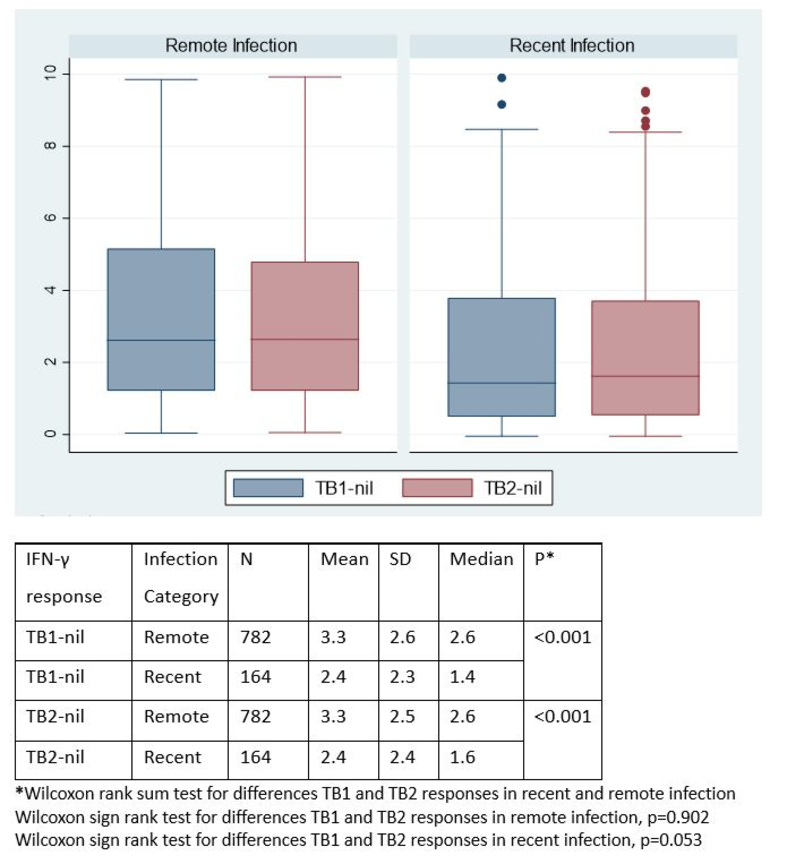

Supplement: S3 Fig — (TIF) [file pgph.0003182.s004.tif]

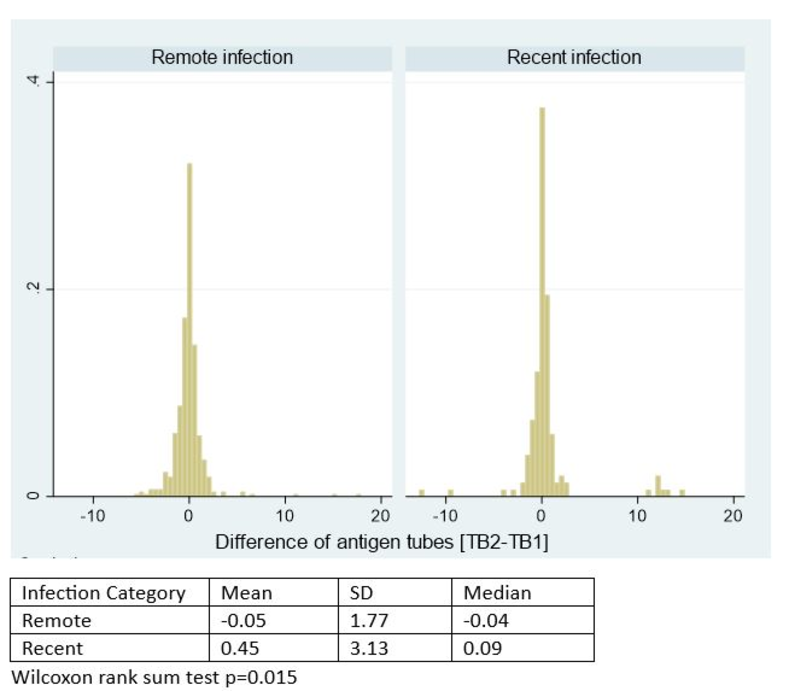

Supplement: S4 Fig — (TIF) [file pgph.0003182.s005.tif]
